# Reproducible biomedical benchmarking in the cloud: lessons from crowd-sourced data challenges

**DOI:** 10.1186/s13059-019-1794-0

**Published:** 2019-09-10

**Authors:** Kyle Ellrott, Alex Buchanan, Allison Creason, Michael Mason, Thomas Schaffter, Bruce Hoff, James Eddy, John M. Chilton, Thomas Yu, Joshua M. Stuart, Julio Saez-Rodriguez, Gustavo Stolovitzky, Paul C. Boutros, Justin Guinney

**Affiliations:** 1 Biomedical Engineering, Oregon Health and Science University, Portland, OR 97239 USA; 2 Sage Bionetworks, Seattle, WA USA; 3 IBM Research, Yorktown Heights, NY USA; 4 Department of Biochemistry and Molecular Biology, The Pennsylvania State University, University Park, State College, PA USA; 5 University of California, Santa Cruz, Santa Cruz, CA USA; 6 Institute for Computational Biomedicine, Heidelberg University, Faculty of Medicine and Heidelberg University Hospital, Bioquant, Heidelberg, Germany; 7 Joint Research Center for Computational Biomedicine, RWTH Aachen University, Faculty of Medicine, Aachen, Germany; 8 Ontario Institute for Cancer Research, Toronto, Canada; 9 Departments of Medical Biophysics and Pharmacology & Toxicology, University of Toronto, Toronto, Canada; 10 Departments of Human Genetics and Urology, University of California, Los Angeles, CA USA; 11 Jonsson Comprehensive Cancer Centre, University of California, Los Angeles, CA USA; 12 Institute for Precision Health, University of California, Los Angeles, CA USA; 13 Biomedical Informatics and Medical Education, University of Washington, Seattle, WA 98195 USA

## Abstract

Challenges are achieving broad acceptance for addressing many biomedical questions and enabling tool assessment. But ensuring that the methods evaluated are reproducible and reusable is complicated by the diversity of software architectures, input and output file formats, and computing environments. To mitigate these problems, some challenges have leveraged new virtualization and compute methods, requiring participants to submit cloud-ready software packages. We review recent data challenges with innovative approaches to model reproducibility and data sharing, and outline key lessons for improving quantitative biomedical data analysis through crowd-sourced benchmarking challenges.

**Electronic supplementary material:**

The online version of this article (10.1186/s13059-019-1794-0) contains supplementary material, which is available to authorized users.

## Introduction

The role of the *algorithm* in biomedical research has been growing steadily, propelled by technological advances in the high-throughput capture of molecular, cellular, and clinical states. The complexity and volume of diverse data types—spanning omics, imaging, and clinical phenotyping—require similarly complex pipelines and algorithms for processing and interpretation. Despite the central role of algorithms in supporting the biomedical research community, mechanisms for their distribution, evaluation, and comparison are lacking. Today, the predominant paradigm for algorithm assessment is self-reporting, a conflict of interest known as the “self-assessment trap” [1]. By definition, self-assessment of an algorithm is highly biased and can mask critical problems such as overfitting, incomplete documentation, software portability, and poor generalizability. These issues collectively impede the successful utilization and translation of algorithms in the lab and the clinic.

**Fig. 1:**
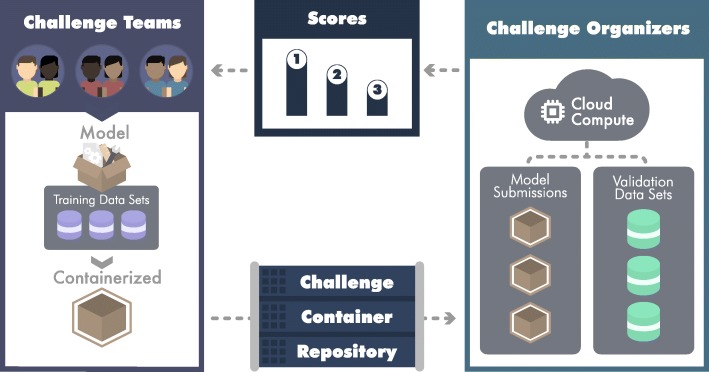
Challenge cycle overview. For each challenge, participants can form teams of one or more individuals. Challenge teams work together to develop a model (depicted as open box), train their model on training data (purple cylinders) provided by the challenge organizers, containerize their model (closed box with outline), and submit their model to the challenge container repository. Submitted models are run on validation data (green cylinders) on a cloud computing system by the challenge organizers. Once predictions produced by the models are evaluated and scored, results are made available to the challenge teams. Teams can use this information to make improvements to their model and resubmit their optimized model

Crowd-sourced data challenges are an increasingly popular mechanism to address the aforementioned shortcomings of method development. Data challenges incentivize teams to work on complex problems, and provide a robust and unbiased framework for assessing the performance of resulting methods [2]. The DREAM Challenges are an example of a data challenge community focused on the rigorous assessment of biomedical tools and algorithms, with over 50 completed challenges over the last decade [3]. As DREAM has evolved with its communities, it has needed to confront a critical problem—many current algorithmic problems cannot be easily evaluated using *open data*. Rather, concerns around data size and privacy are making it increasingly difficult to transfer datasets to participants for their evaluation. To resolve this problem, several alternative forms of data sharing have been explored, and a paradigm described as “model to data” (M2D) has emerged [4] and Fig. 1). In M2D, the underlying dataset remains hidden from users; rather, models are moved to the data for execution and evaluation in protected compute environments. In addition to solving model reproducibility problems, model to data challenges enable assessment of models on future (i.e., prospective) data sets and facilitate *continuous benchmarking* as new models and data sets emerge.

DREAM has now successfully completed several M2D challenges, demonstrating the feasibility and utility of this paradigm. Each M2D challenge has revealed unique logistical and technological hurdles associated with data storage and access, scalability of compute resources, modularity of pipelines and algorithms, and the complexity of training models in a cloud environment. These challenges have also revealed important lessons on how to leverage cloud and virtualization technologies, how to utilize protected and sensitive data, and how to engage communities in solving complex biomedical problems. Here, we review five M2D challenges covering a broad range of scientific questions and data types. We highlight key lessons on benchmarking, challenge execution, model reproducibility, and data sharing. These lessons provide concrete steps for optimizing future cloud-based biomedical data challenges and also serve as a roadmap for creating a distributed benchmarking ecosystem that connects algorithms to data.

## M2D challenges overview

**Fig. 2:**
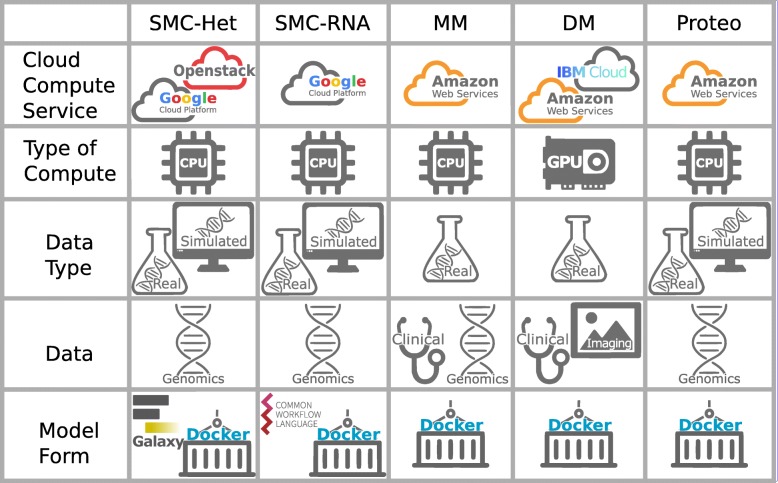
Challenge features. Challenges used cloud computing services for running and evaluating models including Google Cloud Platform, Openstack, Amazon Web Services, and IBM Cloud. Models were designed to run using either CPUs or GPUs. The type of data used in running and evaluation of models was either real data (obtained from patients or cell lines) or simulated using a computer algorithm. Challenges used genomic data, such as DNA sequencing, RNA sequencing, and gene expression; clinical phenotypes; and/or images. Models could be submitted to a challenge in the form of a galaxy workflow, docker image, or CWL (Common Workflow Language) workflow

The M2D challenges examined here address a common problem: how to facilitate the training and evaluation of algorithms on hidden data at scale using cloud resources. This problem is addressed in different ways, depending on the unique technical and scientific constraints of each challenge. The variety of approaches is summarized in Fig. 2 across five areas: (i) cloud environment, (ii) compute requirement, (iii) data generation method, (iv) data type, and (v) form of submitted model (algorithm). Here, we briefly introduce each of the challenges before describing the lessons learned with respect to implementation of the M2D paradigm.

### Digital Mammography Challenge

The Digital Mammography (DM) DREAM Challenge was a data challenge designed to develop and assess algorithms for improved breast cancer detection [5]. The DM Challenge encouraged the use of deep learning methods applied to a large image repository of screening mammograms, with the goal of reducing the ~ 10% false-positive rate of screening mammography [6]. The Challenge asked participants to train and validate models that identify women with breast cancer using a hidden data cohort of screening images and limited demographic information.

The Challenge utilized multiple independent data cohorts for training and validation (see Table 1), with Kaiser Permanente Washington contributing the primary challenge cohort. The condition of use for all images dictated that the *images could not be distributed directly to participants*, thereby requiring the M2D paradigm whereby participants submitted containerized models to challenge organizers. Participants were able to submit three containerized pipelines for handling data pre-processing, model training, and model prediction which were then run by the challenge organizers within protected cloud environments (see Table 2). Given the large data sets and deep learning requirements, computational resources available to participants included access to GPUs and large storage capacity. The Challenge resulted in 57 teams submitting 310 models during the 7 months of the Challenge. These models established the first-ever benchmarks of deep learning methods for detecting cancer from screening mammograms, with results to be published in a forthcoming manuscript.

**Table 1:** Challenge data characteristics

Challenge	Data types	Data cohorts	*N* samples	Size	Open
Digital Mammography	Human clinical Imaging	Kaiser Permanente	80k patients (640k images)	13 TB	No
		MSSM	1k (15k)	.3 TB	No
		Karolinska	69k (663k)	13.2 TB	No
		UCSF	42k (500k)	10 TB	No
		CRUK	7 k		No
		Total	200k (1818k)	36.5 TB	
Multiple Myeloma	Human clinical; gene expr; DNAseq; Cytogenetics	MMRF	797	11 GB	Yes
		PUBLIC	1444	1 GB	Yes
		DFCI	294	76 GB	No
		UAMS	463	6 GB	No
		M2Gen	105	41 GB	No
		Total	3103	135 GB	
SMC-Het		All	76	22 GB	No
SMC-RNA	Simulated; Human clinical; RNA-seq	Training	31	290 GB	Yes
		Test	20	197 GB	Yes
		Real	32	265 GB	No

Data cohorts describe the source of the data used in the challenge. *MSSM* Mount Sinai School of Medicine, *UCSF* University of California San Francisco, *CRUK* Cancer Research UK, *MMRF* Multiple Myeloma Research Foundation, *DFCI* Dana-Farber Cancer Institute, *UAMS* University of Arkansas for Medical Sciences, *Training* synthetically generated data provided to participants, *Test* synthetically generated data held-out data, *Real* cell lines spiked in with known constructs. The number of samples in digital mammography includes the number of patients and the number of images in parentheses. Open indicates whether the data was publicly available to participants

**Table 2:** Summary of models and teams for challenges

Challenge	Cloud platforms	Model format	# of models	# of teams
Digital Mammography	AWS, IBM Softlayer	Docker	310	57 teams
Multiple Myeloma	AWS	Docker	180	71
SMC-Het	ISB-CGC (Google)	Galaxy, Docker	58	31
SMC-RNA	ISG-CGC (Google)	CWL, Docker	141	16
Proteogenomic	AWS	Docker	449	68

Number of participants from each challenge, as well as model types and submission counts

### Multiple Myeloma Challenge

Multiple myeloma (MM) is a cancer of the plasma cells in the bone marrow, and therapeutic strategies and clinical course depend on a complex interplay of clinical and molecular features. Risk-based therapy is becoming standard of care, creating an urgent need for precise risk stratification model to assist in therapeutic decision-making. The MM DREAM Challenge aimed to accelerate the development and evaluation of such risk models. Previous MM risk models using clinical, genomic, and transcriptomic data have been published [7, 8], yet no objective and systematic assessment of these models has been conducted and none of these has yet been adopted for routine clinical use.

The MM Challenge was structured to provide participants access to large and robust data sets for model training, while utilizing unpublished and proprietary data for unbiased model validation. Validation data sets were acquired from commercial and academic entities on the condition that the data sets could not be directly shared with challenge participants. Consequently, teams were required to submit fully trained and Dockerized models that could be applied to these validation data sets, which included combinations of clinical, genomic, and transcriptomic data. Models were then scored according to their ability to predict disease-free survival in multiple patient cohorts. Well-regarded published models based on gene expression or genomic variants were used as state-of-the-art benchmarks, while simpler models based on age and MM stage were used to provide a lower bound on expected performance. The 427 models submitted by 73 teams were compared against these benchmarks and against one another, with the best-performing ones significantly outperforming existing models and identifying novel gene candidates for follow-up studies.

### SMC-Het: ICGC-TCGA Tumor Heterogeneity Challenge

Subclonal reconstruction is the quantification and genotyping of each individual cell population within a tumor. SMC-Het was a global effort to improve methods in this field, including evaluation of the use of somatic variants to identify the different subclones in the sample, assign mutations to these different subpopulations, and reconstruct the evolutionary tree of these subpopulations. To accomplish this, the organizers of this DREAM Challenge created simulated tumors with known tumor evolutionary histories, accepted Docker containers from participants, and scored the methods on new simulated tumors. The methods were able to be rescored as improvements were made to the tumor heterogeneity simulator itself [9].

Participants were provided custom Google Cloud VM images running Galaxy and Planemo to allow them to develop analysis pipelines. Contestants were given examples of the input data, consisting of somatic variant VCF and copy number alteration files, along with the result files. These files were small enough so that they could be packaged on the VM image along with the development software. A copy of the evaluation and scoring code was also packaged as a Galaxy tool. This allowed users to quickly cycle between developing tools and evaluating their results on a set of training files. Once contestants were ready to submit, a submission system was built directly into the VM, accessible via a command-line utility or a website running on the VM. This utility would package the participants Galaxy tools and workflow, as well as extract Docker container images from the VM, and copy them all to Synapse Challenge Platform, before creating a submission entry in the evaluation queue. By the challenge’s close, the organizers received 76 entries from 31 teams.

### SMC-RNA: ICGC-TCGA RNA-Seq Challenge

The transcribed genome serves a multitude of functions within a cell including carrying the information to encode proteins and serving as regulatory components. Coding and noncoding RNA have been demonstrated to play an important role in cancer. Dysregulation of RNA expression and formation of chimeric fusion proteins are both common features in tumor cells. Next-generation sequencing can both quantify RNA abundance and define its structure, allowing simultaneous identification and quantitation of chimeric transcript and protein products not present in normal cells, which can be used as diagnostic markers (e.g., TMPRSS2-ERG in prostate cancer) or drug targets (e.g., BCR-ABL in CML). The SMC-RNA DREAM Challenge was an effort to improve standardization, reproducibility, and accuracy of RNA-Seq methods. Participants were provided Illumina-based RNA sequencing from simulated tumor samples and evaluated on their ability to quantify isoform abundance and to detect chimeric fusion transcripts.

The SMC-RNA Challenge provided participants the flexibility to choose their development environment through either the ISB Cancer Genomics Cloud or Seven Bridges Cancer Genomics Cloud. For participants who used ISB-CGC, the challenge provided access to training data on a Google storage bucket as well as custom Google VM images to use for their development environment. On SBG-CGC, training data was made accessible on a public project that users could clone and use in conjunction with the Seven Bridges Software Development Kit. Training data, which consisted of Illumina-based sequence FASTQ files, was synthetically generated in the same way as testing data. In order to standardize the submissions and evaluation of the methods, participants were required to define a CWL workflow for their tool and package their runtime environment using a Docker container. ISB-CGC participants were responsible for writing their own tool definition and workflow in CWL. The submission process consisted of pushing their Docker container to a public repository and submitting a merged CWL workflow (which references the Docker image) to Synapse. On SBG-CGC, participants were able to utilize the Rabix tool and workflow editors to both describe the tool and string together multiple tools into a workflow. For submission, participants shared a successfully completed task. The evaluation framework consisted of two steps: running submitted methods on test data using ISB-CGC and scoring their performance. The organizers received 76 submissions from 14 teams for fusion detection and 65 from 8 teams for isoform quantification.

### Proteogenomic Challenge

The NCI-CPTAC DREAM Proteogenomics Challenge (Proteogenomics Challenge) aimed to use the community to develop computational tools to predict the proteome and phospho-proteome from genomics and transcriptomics as a means to understand the association between genome, transcriptome, and proteome in tumors. Measuring the proteome is very challenging, but recent rapid technology developments in mass spectrometry are enabling increasing deep and accurate proteomics analysis. The characterization and analyses of alterations in the proteome, such as phosphorylation, provide additional insight into the functionality of proteins and their deregulation in cancer. Collectively, (phospho) proteomic has the promise to shed light into the complexities of cancer and may improve development of both biomarkers and therapeutics. This challenge asked participants to find new methods for imputing missing values in proteomic data, predict protein abundances, and identify phosphorylation events from genomic data.

This Proteogenomics Challenge used public and novel proteogenomic data to answer fundamental questions about how different levels of biological signal relate to one another. The challenge was built using a collection of tumor/normal pairs, with matched genomic, transcriptomic, and proteomic characterization for breast and ovarian cancer, large part of which had not yet been released to the public. Data was provided by the CPTAC (National Cancer Institute’s Clinical Proteomic Tumor Analysis Consortium). Since the novel data could not be directly shared with the challenge participants, teams were required to submit fully trained and Dockerized models that could be applied to this data. The challenge attracted methods from 68 teams with 449 submissions over the three sub-challenges.

## Lessons learned

### Increased demands on participant to construct reproducible models

In traditional challenge formats, participants download test data sets, run their method, and upload the outputs of their models to challenge organizers. While simple and convenient to participants, this format does not take advantage of the considerable strengths associated with M2D that includes the ability (i) to easily disseminate models to the public, (ii) to perform post hoc experiments and new analyses after the closure of the challenge, (iii) to evaluate performance in newly obtained data sets, and (iv) to develop and experiment with ensemble models. Naturally, there is a trade-off with the additional complexity and overhead required to host—and participate in—a M2D challenge compared to a *traditional* data challenge. However, while there is an increased *upfront* burden on participants which may negatively impact participation, this is offset by the greater flexibility and rigor that M2D bring to challenges. However, as familiarity with virtualization and workflow technologies continues to grow—and as the technology itself matures—we expect that these burdens on participants will substantially decrease.

### Importance of designing challenges in conjunction with data contributors

Every benchmarking challenge relies on input datasets, and obtaining unpublished validation data requires close collaboration with researchers generating the data. There may be a number of concerns around access and security of that data. Among these is the desire of data contributors to have the first opportunity to publish key scientific results from their data. This can at times conflict with the need to keep datasets private to ensure an unbiased benchmarking challenge. Additionally, challenge validation data may be composed of multiple cohorts each originating from a separate data contributor, as was the case in the Multiple Myeloma Challenge. In such cases, these data contributors may view each other as competitors, and additional care must be taken to ensure such validation data is protected. To ensure the trust of data contributors, we developed guidelines regarding permissible summary statistics or sample characteristics participants could return and audited these accordingly. To further protect validation data in both the Digital Mammography and Multiple Myeloma challenges, we applied a strict size limit to output logs. To drive method development, participants need easy access to training data with clear information about the “truth.” In many cases, the most viable method is to develop synthetic models to generate training data. For example, in the case of the SMC-RNA Challenge, several rounds were scored using synthetic FASTQ files that could be provided to participants with minimal concerns around data privacy.

### Develop robust strategies for generating training data

The selection of training and debugging data is a complex issue, and each challenge has had to adopt customized approaches depending on data availability. For some challenge data, there were no privacy issues and training data—a subset of the full data set—could be shared directly with participants, as was done for the Proteomics Challenge. Others challenges have used simulated data to bypass these issues—as in the SMC-RNA Challenge. While simulated datasets may not completely recapitulate the underlying biology, they can provide a baseline on known and expected qualities of the data and can assist in developing robust computational pipelines. For the DM Challenge, none of the primary challenge data could be disseminated to participants. To help with model training, challenge participants could submit Dockerized containers that were permitted to train models using a subset of the imaging data. Limited feedback was returned to participants from method logging, but this required careful scrutiny by challenge organizers to ensure no sensitive data was leaked through the returned log files. Many teams in the DM Challenge utilized public datasets for training seed models and then used the private challenge data for further optimization.

### Monitoring, rapid correction, and feedback to participants

A public-facing challenge is a complex interaction that involves providing documentation to users, accepting work products, and making sure outputs are compatible and that novel methods from external parties will function correctly within a pre-set evaluation system. Each of these steps can contain novel software-development, algorithmic, or scientific work. Consequently, challenge procedures need to be put in place that will mitigate common failures that include (1) carefully documenting the input data format and requirements for the model output format, (2) providing a small, representative data set which participants can download and test with their code prior to submission, (3) providing a mechanism for rapid assessment and feedback of execution errors using a reduced size dataset, and (4) performing upfront validation prior to initiating computational expensive and long-running jobs. When running computational models in the cloud, we are asking participants to give up the close, interactive exploration of data they might normally pursue when tinkering with novel algorithmic approaches and to troubleshoot potential defects in their code. In the event that an algorithm fails to execute, providing log files back to the participants may assist in diagnosing and fixing errors. *However, this has the potential to leak data or sensitive information and must be tightly controlled*. Consequently, if log files must be returned to participants, we recommend using simulated or “open” data for testing and troubleshooting models.

### Estimating and managing computational resources

**Fig. 3:**
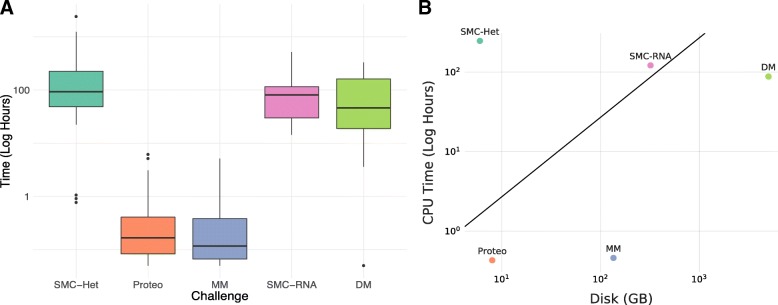
**a**) Distribution of model run times across M2D Challenges. **b**) Comparison between CPU and disk usage among the M2D Challenges. CPU time is in the total wall time for running a single entry against all test samples used for benchmarking. Disk usage is the size of the testing set in GB. The diagonal line represents the point at which the cost of download egress fees and the cost of compute are equivalent. Below the line a M2D approach is theoretically cheaper

For many challenges, computational methods can have non-trivial run times and resource requirements (see Fig. 3). For example in the SMC-RNA Challenge, methods can average 4 h per tumor. When doing the final computational runs, every method submitted needs to be run against every testing set. This can quickly lead to thousands of computational jobs that cost several thousand dollars, all of which is now run at the cost of the challenge organizers. In a number of different challenges, runtime caps had to be put into place to eliminate methods that took multiple days to complete. In the case of the SMC-Het Challenge, methods were limited to a budget of $7/tumor. A high memory machine cost $0.60 an hour, which equated to ~ 12 h of compute time for memory-intensive algorithms. In some challenges, preemptable machines were used for evaluation, because of their lower costs. But these types of VMs work better for short running methods, that can complete before the cloud provider preempt the system. Efforts such as the Digital Mammography challenge, in which both model evaluation and *training* are performed in the cloud, require significantly increased compute resources. In this case, we limited compute budgets to 2 weeks per team per round for model training, with four rounds in the challenge. The high-end GPU servers cost several dollars per hour to rent from cloud providers. Not knowing in advance how many participants would join, we faced the risk of running out of computational resources. From this perspective, it is far less risky to ask participants to provide their own computation but, of course, this is only feasible when data contributors agree to let participants download training data. In short, when organizing a challenge, care must be taken to only commit to run the training phase when it is truly necessary for business reasons, such as sensitivity of training data.

### Increased flexibility to evolve and adapt a challenge over time

During the active phase of the challenge, and even *post* analysis, there is a great deal of additional thought and analysis that goes into the evaluation data and the evaluation criteria. In some cases, there are evaluations that need to be made to the dataset, based on characteristics found during the challenge. Fixing these systems during the running of the challenge is inevitable, but every disruption disincentivizes participants from continuing work on the challenge and may limit the moral authority of the challenge to drive community-evolution. In previous challenges, if there was an issue with the testing data, it was impossible to adjust it and send back to users for new analysis. But with portable code, it becomes possible to modify the testing set, rerun methods, and evaluate. The SMC-Het Challenge faced the problem that there were no well-accepted standards for the scoring of complex phylogenetic relationships in cancer. This created a need for development of new methods for model simulation and scoring [10], and these greatly increase the risk of unexpected errors, edge-cases or performance degradations. Because the participants submitted reproducible code, their methods could be reevaluated using newly generated models and evaluation methods.

### Model distribution and re-use

Docker containers have a very modular format for distribution, and there exist several different repositories that allow for users to download the software image with a single command. However, this is only one component of distribution; there is also a need for systems that document how to invoke the tool, with descriptions of command-line formatting, tunable parameters and expected outputs. If these descriptions are machine parseable, they can be deployed with workflow engines that manage large collections of tasks. In the case of SMC-Het, the chain of commands was documented using the standards from the Galaxy Project [11]. For the SMC-RNA Challenge, these descriptions were made using the Common Workflow Language (CWL) [doi:10.6084/m9.figshare.3115156.v2]. These systems allow for automated deployment, and are used as part of the evaluation framework deployed by challenge organizers. Because of this, two of the winning methods from the SMC-RNA Fusion calling challenge have been integrated into the NCI’s Genomic Data Commons [12] (GDC) standard analysis pipeline, and are now being applied to a number of datasets including TARGET, CPTAC, MMRF and TCGA.

## Future of data challenges and cloud-centric analysis

The purpose and scope of data challenges are quickly evolving in response to a rapidly maturing compute ecosystem, the growing popularity of challenges to solve complex problems, and the use of challenges to demonstrate and advertise technical competencies. Most importantly, challenges provide a robust and unbiased mechanism for assessing the best approach to solving quantitative problems. This is increasingly important in a world where algorithms are playing critical roles in biomedical decision making. The ability to objectively track the performance of algorithms over time - across a wide array of data cohorts - can play an important role in establishing confidence that algorithms are achieving their purported goals. Below, we outline some of the innovative and exciting directions for future data challenges, and biomedical analysis more broadly.

### Bridging the translation gap

One key bar algorithm developers need to pass to induce their tool or algorithm to be broadly adopted is *believability*: does the algorithm achieve its purported claims. In this regard, a bottleneck in most of biomedicine is not the lack of algorithms, but instead the lack of *validated and verified* algorithms. This lack of validation is a major contributor to the failure of tools to move beyond the research setting into a context that can more directly impact human health (i.e., the translational gap). Data challenges solve this problem by developing benchmarks and objective standards for tool evaluation. Challenges reveal the strengths and weaknesses of competing approaches to solving domain-specific problems, and in doing so, can accelerate the selection and adoption for tools to use in the lab and the clinic. Utilizing the M2D approach, the ability to capture methods and replay them in a controlled environment provides the opportunity to close the gap to direct patient care.

### Distributed benchmarking ecosystem

Some of the most highly impactful biomedical data is not readily shareable due to concerns around privacy, personal health information, or intellectual property risks. Well-known examples of such data include clinical trial data, electronic healthcare records (EHR), and genetic data. The inability to access these critical datasets further contributes to the translational gap. We can imagine, and are developing toward, a frictionless benchmarking ecosystem whereby algorithms are regularly distributed to private clouds and protected data repositories for evaluation on hidden data. Such a system would enable real-time assessment of an algorithm’s performance, and allow this performance to be tracked over time as new data becomes available. Moreover, by distributing an algorithm over many such repositories, differences in performance as a result of collection biases or population differences could be assessed, and be used to determine an algorithm’s generalizability. Indeed, DREAM has already begun piloting such approaches with the recently launched EHR DREAM Challenge [13], which will allow participants to develop and assess predictive clinical algorithms across multiple healthcare systems’ data repositories. We intend to use this Challenge to demonstrate the feasibility and value of a secure and distributed benchmarking system.

### Enabling a cloud-centric future for biomedical research

As the rapid expansion of data generation continues, research projects will be increasingly reliant on distributed cloud-based systems for data processing and analysis. Solutions that involve a single lab distributing a package of tools and documentation for running on a single dataset or running a low throughput web server will not scale. Without standards for packaging and documenting how to invoke tools, the frictional cost of transferring software slows down the movement of methods into new cloud resources. Analytical methods need to be packaged using modern cloud-based solutions so that new methods can be quickly moved to new data and deployed by new groups. M2D encapsulates this shifting paradigm, where algorithms are brought to data in a systematic and scalable way. As this paradigm becomes more widely implemented—not only for data challenges but as the predominant architecture for biomedical and genomic data hosting and *data commons*—we envision a future in which the barriers between algorithms and data are substantially reduced, thereby accelerating biomedical insights and applications.

### Conclusion

As the role of algorithms and software tools within the biomedical sciences grows, there is a concomitant need to rigorously evaluate and benchmark their performance. By utilizing cloud-based infrastructure and virtualization software, this is achievable like never before. The data challenges described herein are proof-of-concepts successfully demonstrating how large, complex, and sensitive biomedical data can be used to address scientific questions and benchmark methods. These challenges have also presented an alternative paradigm with respect to data access, algorithm reproducibility, community participation, and objective evaluation. As cloud platforms expand their services at ever cheaper costs, and as biomedical institutions improve federated and integrated capabilities across sites, data challenges and algorithm benchmarking are likely to become important fixtures in the biomedical landscape.

## Additional file

Additional file 1: Review history. (DOCX 18 kb)

## Data Availability

In the case of SMC-Het, the chain of commands was documented using the standards from the Galaxy Project [11]. For the SMC-RNA Challenge, these descriptions were made using the Common Workflow Language (CWL) [doi:10.6084/m9.figshare.3115156.v2]. The workflows for the SMC-RNA Challenge can be found at https://github.com/smc-rna-challenge/ [14].

## References

[1] Norel R, Rice JJ, Stolovitzky G (2011). The self-assessment trap: can we all be better than average?. Mol Syst Biol.

[2] Bender E (2016). Challenges: crowdsourced solutions. Nature..

[3] Saez-Rodriguez J, Costello JC, Friend SH, Kellen MR, Mangravite L, Meyer P (2016). Crowdsourcing biomedical research: leveraging communities as innovation engines. Nat Rev Genet.

[4] Guinney J, Saez-Rodriguez J (2018). Alternative models for sharing confidential biomedical data. Nat Biotechnol.

[5] Trister Andrew D., Buist Diana S. M., Lee Christoph I. (2017). Will Machine Learning Tip the Balance in Breast Cancer Screening?. JAMA Oncology.

[6] Sprague BL, Arao RF, Miglioretti DL, Henderson LM, Buist DSM, Onega T (2017). National performance benchmarks for modern diagnostic digital mammography: update from the Breast Cancer Surveillance Consortium. Radiology..

[7] Shaughnessy JD, Zhan F, Burington BE, Huang Y, Colla S, Hanamura I (2007). A validated gene expression model of high-risk multiple myeloma is defined by deregulated expression of genes mapping to chromosome 1. Blood..

[8] Kuiper R, Broyl A, de Knegt Y, van Vliet MH, van Beers EH, van der Holt B (2012). A gene expression signature for high-risk multiple myeloma. Leukemia..

[9] Salcedo A, Tarabichi M, Espiritu SMG, Deshwar AG, David M, Wilson NM, et al. Creating standards for evaluating tumour subclonal reconstruction. bioRxiv. 2018:310425 [cited 2018 Jul 23]. Available from: https://www.biorxiv.org/content/early/2018/07/15/310425.

[10] Boutros PC, Salcedo A, Tarabichi M, Espiritu SMG, Deshwar AG, David M, et al. Creating standards for evaluating tumour subclonal reconstruction. bioRxiv. 2018:310425 [cited 2018 Jul 12]. Available from: https://www.biorxiv.org/content/early/2018/04/30/310425.

[11] Afgan E, Baker D, van den Beek M, Blankenberg D, Bouvier D, Čech M (2016). The Galaxy platform for accessible, reproducible and collaborative biomedical analyses: 2016 update. Nucleic Acids Res.

[12] Jensen MA, Ferretti V, Grossman RL, Staudt LM (2017). The NCI Genomic Data Commons as an engine for precision medicine. Blood..

[13] EHR DREAM Challenge [Internet]. Available from: https://www.synapse.org/#!Synapse:syn18405991/wiki/589657

[14] Ellrott K, Buchanan A, Creason A, Mason M, Schaffter T, Hoff B, Eddy J, Chilton JM, Yu T, Stuart JM, et al, Reproducible biomedical benchmarking in the cloud: lessons from crowd-sourced data challenges. Source code. Github https://github.com/smc-rna-challenge/. 10.1186/s13059-019-1794-0 PMC6737594 31506093

