# Supplementary material for: Reproducible biomedical benchmarking in the cloud: lessons from crowd-sourced data challenges
Source: Genome Biol. 2019 Sep 10;20:195. doi: 10.1186/s13059-019-1794-0 (PMC6737594; doi:10.1186/s13059-019-1794-0)
Supplement: Supplementary file 1 — Review history. (DOCX 18 kb) [file 13059_2019_1794_MOESM1_ESM.docx]

Review History

**Comments to author and authors’ point-by-point responses to the reviewers’ comments:**

**Reviewer 1**

This manuscript describes the logistics and lessons obtained from the Dream Challenge data analysis competitions, especially about the need to run prediction models on Challenge organizers' computers to protect data privacy and minimize self-reporting bias of the prediction performance. The authors list five Challenge projects to illustrate various practical considerations and lessons, which should be of broad interest and be useful guidance to data providers, algorithm researchers and clinical users participating in the Challenge.

Comments:

From Figure 1, the training data is still transferred to algorithm developers' computers, causing possible privacy issues and data format incompatibility when the models are tested on the validation data. Are there better solutions?

*RESPONSE: The reviewer raises an important point regarding access and use of training data. In some Challenges, we provide training data directly to participants to download and train models. In other Challenges, we have no training data but point participants to public resources that can be used to develop models. In more limited circumstances, we have provided training data that could not be directly accessed by participants.*

*An example is the Digital Mammography Challenge, where we partitioned 50% of the total Kaiser cohort into a training set. However, teams had to submit Docker algorithms that performed all training in a private cloud. In this Challenge, some participants opted to use public datasets to generate initial models, and then utilized the Kaiser training data to further optimize their models. We have updated the “Lessons Learned” section to highlight some of the complexities in generating training/debugging data sets, and the different strategies various challenges have utilized.*

**Reviewer 2**

I am providing my assessment of the submitted manuscript according to the following description of Genome Biology Open Letter: “ … Open Letters provide a forum for the announcement of projects, resources and position statements that are of exceptional interest to the genomics community…”

In this manuscript, a group of authors from Sage Bionetworks described their past experience hosting five DREAM challenges. What’s unique about these challenges is the “Model to Data” structure, meaning the data is hidden and the participants submit their models. In the first half of the paper, they gave a brief overview for each DREAM challenge, including Digital Mammography, Multiple Myeloma, SMC-Het: ICGC-TCGA Heterogeneity, SMC-RNA, and Proteogenomic challenge. The results of these five Challenges have been extensively described before with the release of results. For example, Sprague et al. has described the Mammography challenge in great details.

In the second half of the paper, the authors described the lessons learned hosting these Challenges. As a computational biologist myself who has participated in previous DREAM Challenges, I don’t find this paper interesting and helpful at all, at least not in the current state.

A key question for the authors to think: who are the readers for this open letter? Is this letter written for the people who want to host DREAM Challenge? Or is it for the people who might be interested in participating the Challenge? Maybe it’s for people who don’t know what Dream challenge is. In the introduction section, the authors scratched surface a little bit, but they didn’t stress enough why we need M2D models. It seems to me, the biggest advantage is the patient privacy issues. It seems the five challenges hosted by Sage are all related with patient samples, whose data cannot be released to the public.

*RESPONSE: We are happy to learn of your participation in previous Challenges, and hope that your experience was enriching. You raise an excellent point as to the targeted readership of our manuscript. While those who are hosting challenges will derive the greatest benefit from our manuscript, we believe that the paradigm of M2D is generally useful and should appeal broadly to the research community. Specifically, the following three major groups of readers are our intended targets:*

*(a) those concerned about objective standards and benchmarks within the biomedical and genomics fields*

*(b) those with access to datasets that cannot be broadly shared, but could be critical to establish benchmarks*

*(c) those concerned about the reproducibility and dissemination of methods.*

*The Model-2-Data paradigm solves these 3 aspects, not only in the context of a Challenge, but more generally as models are moved to private clouds hosting protected data.*

I still believe there is advantage of M2D over traditional data download, the authors didn’t make their case here. For example, the first lesson they learned is “Increased demands on participant to construct reproducible models,” a few concrete example will be much more helpful. I also don’t see a “increased demand” for M2D, based on the description here. For example, how many DREAM *challenges are “traditional” and how many are M2D?*

*RESPONSE: We have now successfully completed 8 M2D Challenges since introducing it just 3 years ago.*

*DREAM is in the process of moving away from “traditional” Challenges to M2D Challenges, and we anticipate that eventually all Challenges will be M2D given the benefits described in the manuscript. Indeed, current Challenges in preparation are all M2D Challenges. These include (i) ImmunoOncology challenge to predict response to checkpoint blockade using Phase III clinical trial data (ii) Challenge to predict clinical phenotypes using Electronic Healthcare Medical data (iii) Challenge to predict severity of rheumatoid arthritis from MRI scans (iv) Natural Language Processing Challenge to improve extraction of clinical phenotypes from unstructured physician notes. These Challenges all require M2D as they utilize some portion of data that cannot be directly shared with participants. As these Challenges are all under active development and not publicly announced, we cannot include them in the manuscript itself. However, as DREAM organizers, we believe that M2D will become the predominant paradigm not only for data challenges, but will have strong impacts on conceptions of data sharing.*

The last section is about the “Future of data challenges and cloud-centric analysis.” Rather than describing the excitement of the future, the authors continued to talk about "standard development" and "Continuous Benchmarking." These two sections are technical and do not belong here. I think the authors missed a gold opportunity to demonstrate their vision for the future of cloud-centric analysis. What is the future of data challenge? How it will be used to make an impact in research and in human health?

*RESPONSE: We are grateful for this insightful remark, and have revised our “Future of data challenges” to reflect - what we believe - is an exciting future built on an ecosystem of connected nodes in the cloud that can facilitate more automatic and real-time assessments on data that obviates the onerous negotiations around MTAs and DUAs. Indeed, we have completely written the last section of our manuscript to highlight the importance of the M2D paradigm, and its likely impact in translational science and data ecosystems. The subsections include (i) “Bridging the Translation Gap” - description of how M2D can accelerate translational medicine (ii) “Distributed Benchmarking Ecosystem” - description of how M2D to by used to develop data networks for benchmarking algorithms (iii) “Enabling a cloud-centric future for biomedical research” - description of how M2D represents a paradigmatic shift in how models are developed and disseminated. We believe these sub-sections capture the opportunity and excitement of this paradigm.*

As an open letter, the current writing failed to appeal to this reviewer as a “statements that are of exceptional interest to the genomics community.” I think there are many exciting projects going on in the genomics field that can benefit from cloud-centric analysis. The authors need to significantly rewrite the last section of this paper.

*RESPONSE: In addition to re-writing the last section of our manuscript to capture the excitement of the M2D paradigm and its likely impact in biomedical and genomic research (see previous comment), we have also added a description of how the winning models from the SMC-RNA challenge were transfer to the NCI's GDC for application to a larger number of samples from various projects. We believe this illustrates how capturing re-rerunnable algorithms through our Challenges has facilitated their dissemination and future application.*
